# Association of a genetic variant in the angiopoietin-like protein 4 gene with metabolic syndrome

**DOI:** 10.1186/s12881-019-0825-8

**Published:** 2019-06-04

**Authors:** Sara Kharazmi-Khorassani, Jasmin Kharazmi-Khorassani, Azam Rastegar-Moghadam, Sara Samadi, Hamideh Ghazizadeh, Maryam Tayefi, Gordon A. Ferns, Majid Ghayour-Mobarhan, Amir Avan, Habibollah Esmaily

**Affiliations:** 1 Department of Biology, Varastegar Medical University, Mashhad, Iran; 2 Department of Modern Sciences and Technologies, Faculty of Medicine, Mashhad University of Medical Sciences, Mashhad, Iran; 3 Brighton & Sussex Medical School, Division of Medical Education, Falmer, Brighton, Sussex, BN1 9PH UK; 4 Metabolic syndrome Research center, Mashhad University of Medical Sciences, Mashhad, Iran; 5 Student Research Committee, Faculty of Medicine, Mashhad University of Medical Sciences, Mashhad, Iran; 6 Social Determinants of Health Research, Center, Mashhad University of Medical Sciences, Mashhad, Iran

**Keywords:** Metabolic syndrome, ANGPTL4, rs116843064

## Abstract

**Background:**

Metabolic syndrome (MetS) is characterized by a clustering of cardiovascular risk factors that include: abdominal obesity, dyslipidemia, hypertension and glucose intolerance. Angiopoietin-like protein 4 (ANGPTL4) is a circulating peptide that is an inhibitor of lipoprotein lipase, a key enzyme in lipid metabolism. The objective of this study was to investigate the association of ANGPTL4 gene variants (E40K) with fasting serum triglyceride levels and with cardiovascular risk factors, that included the presence of MetS in 817 subjects recruited from the Mashhad stroke and heart Atherosclerosis Disorders (MASHAD) cohort Study.

**Method:**

ANGPTL4 genotypes were determined using a TaqMan genotyping based real time PCR method. The association of the genetic variant with the risk of metabolic syndrome and its relationship with lipid profile were determined.

**Result:**

The frequency of GG, GA and AA genotypes were 96.9, 2.7 and 0.4% in individuals with MetS, and 78.8, 20.8, 0.4%, in those without MetS. The GA genotype of the rs116843064 polymorphism was associated with a lower risk for MetS (e.g., OR in Codominant genetic model: 0.14, 95% CI: (0.06–0.33), *p* < 0.0001). Subject with an A allele had a higher risk for MetS (OR: 6.72, 95% CI: (3.05–14.82), p < 0.0001). There was a significant difference in fasted lipid profiles across the genotypes for ANGPTL4. Carriers of the AG genotype had higher levels of serum HDL-cholesterol (HDL-C) and lower TG, compared to the GG homozygotes genotype.

**Conclusion:**

The G allele at the rs116843064 polymorphic locus of the ANGPTL4 gene was associated with a lower prevalence of MetS.

## Background

Metabolic syndrome is increasing globally, and is important because of its association with increased cardiovascular disease (CVD) risk [1]. It affects about 20 to 25% of the adult population [2]. MetS is characterised by a clustering of CVD risk factors that include: high blood pressure, dyslipidemia, impaired glucose tolerance and abdominal obesity [1]. MetS is associated with an increased risk of CVD and diabetes mellitus [3, 4]. MetS is also associated with other co-morbidities including fatty liver [5, 6], asthma [7, 8] and some cancers [9–11].

The development of MetS depends on environmental (e.g., physical inactivity, diet, gender, age) and genetic factors [12–16]. Chronic stress has also been shown to increase the risk of MetS [17]. Angiopoietin–like proteins are secreted proteins [18] that have major role in the metabolism of energy, lipid and glucose [19, 20]. Angiopoietin–like proteins 4 (ANGPTL4) is a circulating peptide which inhibits lipoprotein lipase [21] and hence influences the metabolism of triglyceride-rich lipoproteins [22, 23].

Long periods of fasting, low calorie diet and a diet high in fat and energy increase plasma free fatty acid concentrations and levels of ANGPTL4, most probably by activation of PPARs in liver and adipose tissue [24, 25]. ANGPTL4 is abundantly expressed in the liver, placenta and adipose tissues [26]. T266 M (rs1044250) and E40K(rs116843064) are two variants of the ANGPTL4 gene [21]. E40K is a fairly rare variant that is found in about 3% of the population of European ancestry [27] and is associated with lower serum LDL-cholesterol and triglyceride and higher HDL-cholesterol [27, 28].

However, other reports have suggested that the serum ANGPTL4 is positively associated with serum triglycerides in individuals with MetS [29]. These variants are also associated with CVD. Abid et al. found that in CAD subjects the GA genotype for rs116843064 polymorphism there was an increased CVD risk [21]. It has also been reported that serum ANGPTL4 is lower in subjects with type 2 diabetes. However no associations were found in subjects with MetS or obesity [30], although subjects with MetS have higher levels of plasma ANGPTL4 [31]. A study in knock-out mice observed that Angptl4-deficient mice have altered lipid metabolism and also smaller atherosclerotic lesions [32]. The primary aim of this current study was to evaluate the association between the rs116843064 gene variant of the ANGPTL4 gene and the presence of MetS.

## Methods

### Study population

The study population comprised 260 with, and 557 individuals without MetS, who were recruited from the Mashhad Stroke and Heart Atherosclerosis Disorder (MASHAD) cohort study. They were recruited through a cluster-randomized-recruitment in 2007–2008, as described previously [33, 34]. MetS was defined according to the International-Diabetes-Federation (IDF) criteria including waist circumference > 94 cm in men and > 80 cm in women along with at least two of the other criteria for MetS, such as fasting serum triglycerides> 150 mg/dl, HDL-C < 40 mg/dl in men or < 50 mg/dl in women, BP > 130/85 mmHg and fasting glucose> 100 mg/dl. In the current study subjects with CAD, stroke and peripheral arterial disease were excluded.

Informed written consent was obtained from all individuals, and the study protocol was approved by the Ethics Committee of the MUMS. All participants were able to read and understand and were willing to provide written, informed consent.

General entry and exclusion criteria for this study and public specifications of the sample participants, including marital status, occupational status, drug use and biochemical measurements were obtained as previously described (32–33).

### Anthropometric and biochemical measurements

Anthropometric parameters containing weight (kg), height (m), waist circumference (cm), hip circumference (cm), waist/hip (cm), BMI (kg/m^2^), systolic blood pressure (mmHg) and diastolic blood pressure (mm Hg) were determined for all participant as previously described [34]. To evaluated BMI body weight (kg) divided by squared height in meters (m^2^) was used. Biochemical parameters including fasted serum triglyceride(mg/dl), cholesterol (mg/dl), HDL-C(mg/dl), fasting blood glucose (mg/dl), serum Hs-CRP (mg/dl) and uric acid were measured as explained previously [35].

### Genotyping

Genomic DNA was extracted from peripheral blood using a QIAamp-DNAMini-Kit (Qiagen, SanDiego, CA) and the manufacturer’s protocol. Using the NanoDrop®-1000-Detector (NanoDrop-Technologies, Wilmington, USA) the purity and concentration of DNAs were evaluated. The genotyping of the ANGPTL4 rs116843064 polymorphism was performed using Taqman®-probes-based assay. The PCR reaction was performed in a total volume of 12.5 μl through 10 ng DNA in TaqMan® Universal Master Mix with particular primes and probes (Applied Biosystems Foster City, CA). ABIPRISM tools with SDS-version 2.0 software has been used to examine the content of allelic samples.

### Statistical analyses

The analysis of the data was performed using SPSS 22 software (SPSS Inc., Chicago, IL). A Kolmogorove Smirnov test was untaked to determine the normality of the data within groups. The genotype and allele frequencies of the ANGPTL4 rs116843064 polymorphism were determined for analysis for the Hardy–Weinberg equilibrium (HWE) through Pearson χ distribution. Kruskal-Wallis and Mann-Whitney U tests, or ANOVA and t tests were used to evaluate the differences between groups. The relationship between the risk of MetS with GG and GA genotypes, versus the AA genotype under recessive genetic model, were investigated through various genetic models. MetS risk was expressed as the odds ratio (OR) and its corresponding 95% confidence interval (CI). The analyses were two-sided and statistical significance was set at *P* < .05.

## Results

### General characteristic of subjects

Anthropometrics and biochemical characteristic of MetS and healthy subjects are presented in Table 1. The mean age of the individuals with MetS and non-MetS subjects were 49.53 ± 7.64 and 49.59 ± 8.54, respectively. BMI, waist circumference, weight, hip circumference, waist/hip, systolic blood pressure and diastolic blood pressure, fasting serum triglycerides, uric acid and Hs-CRP were higher than in the control group (*p* < 0.05). Also fasting blood glucose was higher in MetS patients but there was no significant difference between the two groups.

**Table 1:** Anthropometrics and biochemical data of MetS and healthy group

	Non-MetS	MetS
Sex		
Woman (%)	340(61.0%)	140(53.8%)
Men (%)	217(39.0%)	120(46.2%)
Total (%)	557(100%)	260(100%)^*^
Age (y)^a^	49.59 ± 8.54	49.53 ± 7.64
BMI (kg/m^2^)	27.55 ± 4.62	29.57 ± 4.12^*^
Waist circumference (cm)	94.84 ± 11.57	100.42 ± 9.01^*^
Height (m)	1.60 ± 0.09	1.62 ± 0.09
Weight (kg)	70.84 ± 12.61	78.17 ± 11.15^*^
Hip circumference (cm)	102.72 ± 9.23	106.30 ± 8.49^*^
Waist/hip (cm)	0.92 ± 0.08	0.94 ± 0.07^*^
SBP (mmHg)	123.16 ± 20.27	127.75 ± 19.45^*^
DBP (mm Hg)	79.09 ± 11.43	82.15 ± 11.08^*^
Serum Triglyceride(mg/dl)^b^	116.00 (91)	189(103) ^*^
Serum Cholesterol (mg/dl)	191.05 ± 40.32	186.78 ± 41.27
FBG (mg/dl)	96.76 ± 43.04	102.51 ± 48.94
HDL(mg/dl)	42.64 ± 10.90	35.71 ± 10.59^*^
LDL(mg/dl)	115.65 ± 34.17	101.35 ± 36.91^*^
Uric acid	4.59 ± 1.44	5.08 ± 1.57^*^
Hs-CRP (mg/dl)^b^	1.61 (2.25)	1.78(2.80) ^*^

^a^Data are presented as mean SD

^b^Data for serum Triglyceride, Hscrp are reported as med (IQR)

*Abbreviation*: *BMI* body mass index, *SBP* Systolic blood pressure, *DBP* Diastolic blood pressure, *TG* triglyceride, *HDL* high density lipoprotein, *HsCRP* high sensitive CRP

* = *P* 0.05>

However height, serum cholesterol, HDL and LDL were higher in the non-MetS group (Table 1).

### Clinical characteristic of populations

We have found that the genotype frequency of GG, GA and AA were 96.9, 2.7 and 0.4% in MetS group and 78.8, 20.8, 0.4%, respectively in those without MetS (Table 2). The distribution of genotypes and allele frequencies of ANGPTL4 gene rs116843064 polymorphism, were in Hardy-Weinberg equilibrium (HWE) (*P* < 0.05).

**Table 2:** Distribution of genotypes and allele frequencies and their association with metabolic syndrome

SNP		Total	Non-MetS	MetS	Odds ratio(95% CI)	*P* Value
Genetic models		817(100%)	557(100%)	260(100%)		
Codominant	GG	691(84.6%)	439(78.8%)	252(96.9%)	Ref Cat 1	
	AG	123(15.1%)	116(20.8%)	7(2.7%)	0.10(0.04–0.22)	< 0.0001
	AA	3(0.4%)	2(0.4%)	1(0.4%)	0.87 (0.07–9.65)	0.99
HWE		< 0.05	< 0.05	< 0.05		
	A	68(5.5%)	61(8.4%)	7(1.3%)	6.72(3.05–14.82)	< 0.0001
	G	1178(94.5%)	665(91.6%)	513(98.7%)	Ref Cat 1	

Logistic regression analysis adjusted for age and sex

*Ref Cat* reference category, *CI* confidence interval, *HWE* Hardy–Weinberg equilibrium

Logistic regression analysis was used to calculate association of polymorphisms and metabolic syndrome

### Association of the genetic variant with MetS

The distribution of the ANGPTL4 gene rs116843064 polymorphism genotypes were investigated in genetic various models (Table 2). These data indicated that the GA genotype of the rs116843064 polymorphism in Codominant model was associated with a lower risk for MetS (e.g., OR in Codominant genetic model: 0.14, 95% CI: (0.06–0.33), *p* < 0.0001). Also subjects with an A allele had a higher risk for MetS (OR: 6.72, 95% CI: (3.05–14.82), *p* < 0.0001).

### Association between rs116843064 polymorphism and lipid profile

The relationship between the ANGPTL4 gene rs116843064 polymorphism and serum fasted TG and HDL level in total population and non-MetS group is presented in Table 3. This shows that the carriers of GA genotype had a higher level of serum HDL and lower serum TG, compared with the GG homozygotes, with the wild genotype (*p* < 0.0001).

**Table 3:** Association between SNP and serum TG and HDL level

Genetic model		HDL serum level (mg/ml)	*P*.value	TG serum level (mg/ml)	*P*.value
Total population	AA	28.50 ± 4.95	0.36	157.50(125–190)	0.75
	AG	44.67 ± 12.07	< 0.0001	108(81–132.50)	< 0.0001
	GG	39.36 ± 10.89	Ref.1	156(104–226)	Ref.1
MetS	AA	34.30 ± −	0.60	468(.)	0.03
	AG	33.10 ± 11.36	0.38	174(131–345)	0.86
	GG	35.78 ± 10.61	Ref.1	189(154–254)	Ref.1
Non-MetS	AA	28.50 ± 4.94	0.17	157.50(125-.)	0.58
	AG	45.71 ± 11.28	0.009	100(71–125.25)	0.005
	GG	41.88 ± 10.64	Ref.1	122.50(88–184.75)	Ref.1

Ref cat: reference category, General linear model was used to calculate association of genotype and lipid profile serum level. Mean ± SD and median(IQR) was used to report HDL and TG levels

Logistic regression analysis Adjusted for age and sex

## Discussion

Multiple studies have revealed that metabolic syndrome is a risk factor for diabetes mellitus, hypertension, pro-inflammatory state, central adiposity, dyslipidemia and high concentrations of ANGPTL4 [18, 36]. A large number of studies have shown that the E40K variant within the *ANGPTL4* gene is linked with significant differences in serum triglycerides and HDL cholesterol. This relationship can be explained at least in part by its function in the inhibition the activity of lipoprotein lipase and stimulates adipose tissue lipolysis and relates to dyslipidemia [23, 24]. A meta-analysis comprising 49,549 subjects has shown that the rs116843064 SNP is a missense variant in ANGPTL4 gene that is involved in determining triglyceride concentrations [37]. Genotypes of SNPs in 1654 individuals of Chinese Han population verified the potential function of the ANGPTL4 variants on circulating lipid levels and diseases that are related to atherosclerosis [38].

Free fatty acids through the lipid-sensing peroxisome proliferator-activated receptors (PPARs) play a main role in the expression regulation of *ANGPTL4* [18]. On the other hand a significant correlation is found between the ANGPTL4 concentration and FFA levels in subjects with type 2 diabetes mellitus (T2DM) compared to healthy individuals [39].

Sadeghabadi et al., have reported an association of plasma levels of ANGPTL4 and expression of PPARγ gene with metabolic characteristics in obese children and adolescents. Although both of PPARγ gene expression and levels of ANGPTL4 were decreased in individuals with obesity, significant association were not found in obese children with insulin resistance as compared to those without insulin resistance and also in obese children with or without metabolic syndrome [40]. Although the association of ANGPTL4 variant and risk of MetS has been inconsistent, the results of some recent investigations are in line with the findings of the current study. The results of the current study indicated that carriers of the A allele (AA or GA genotypes) of the *ANGPTL4* gene have higher levels of HDL but lower TG levels, compared to those with GG homozygote genotype. Moreover the GA genotype was associated with reduced risk of MetS. Some other studies showed that the E40K variant was linked with lower serum TG and higher HDL-cholesterol [27, 28]. The Atherosclerosis Risk in Communities study and the Copenhagen City Heart Study demonstrated that the E40K variant was associated with a lower levels of TG and LDL cholesterol but higher levels of HDL cholesterol [28]. In line with these findings, Dewey et al. showed that carriers of the E40K and other inactivating mutations in ANGPTL4 had lower levels of triglycerides and also a lower risk of coronary artery disease compared with non-carriers. The authors of this study found that the presence of the E40K variant was associated with a 19% lower risk of coronary artery disease than for E40 homozygotes [41]. Experiments on the regulation of Angptl4 expression within arterial macrophages have shown that formation of foam cells is suppressed by Angptl4 expression and leads to a lower risk of atherosclerosis. Moreover, overexpression of Angptl4 may play a crucial role in lipid metabolism [42]. Abid et al. speculated that the variants of ANGPTL4 may be associated with the reduced fasting triglyceride levels and consequently these variants may reduce the risk of cardiovascular disease [21]. A prospective study conducted by Folsom and colleagues revealed that individuals with E40K variant possess reduced genetic risk for coronary heart disease [43]. Stejskal et al., demonstrated that in individuals with MetS the concentration of serum ANGPTL4 was directly associated with serum TG [29]. Smart-Halajko et al., showed an inverse association between serum ANGPTL4 and HDL-cholesterol and could not find any significant associations between coronary heart disease and the levels of serum ANGPTL4 [44]. An investigation of the association between ANGPTL4 levels and metabolic parameters in a population based study, concentrations of ANGPTL4 were found to be positively related to the metabolic syndrome, and plasma ANGPTL4 concentrations were inversely related to LDL-C and HDL-C, but positively associated with serum triglycerides [45]. Other study showed that polymorphism within the ANGPTL4 gene is not related to metabolic components in white subjects [46].

Miida et al. observed that in individuals with MetS, serum ANGPTL4 was directly associated with serum triglycerides [31]. Muendlein et al., have shown that the variants of ANGPTL4 and the plasma levels of ANGPTL4 may have a potential role in the prediction of cardiovascular events and also the characteristics of the MetS were linked with the levels of serum ANGPTL4 [26]. However, Dlouha et al., did not find a relationship between the rs116843064 ANGPTL4 gene polymorphism and risk of acute coronary syndrome in a Czech population [39]. The results of a cohort study by Staiger et al., demonstrated that fasting FFA and adipose tissue lipolysis were directly associated with plasma ANGPTL4 [36]. The results of various studies indicate that plasma levels of ANGPTL4 were higher in MetS and T2DM compared to the control group [18, 30].

## Conclusion

In summary our results supports a significant relationship between the rs116843064 genetic polymorphism within the ANGPTL4 gene with the presence of MetS and lipid profile.

## Abbreviations

ANGPTL4: Angiopoietin-like protein 4
BMI: Body mass index
CAD: Coronary artery disease
CVD: Cardiovascular disease
DBP/SBP: Diastolic/systolic blood pressure
FBG: Fasting blood glucose
HC: Hip circumference
HDL: High density lipoprotein
Hs-CRP: High sensitivity C reactive protein
HWE: Hardy-Weinberg equilibrium
IDF: International Diabetes Federation
IQR: Inter quartile range
LDL: Low density lipoprotein
MASHAD: Mashhad Stroke and Heart Atherosclerosis Disorder
MUMS: Mashhad University of Medical Science
PCR: Polymerase Chain Reaction
SD: Standard deviation
SNP: single nucleotide polymorphism
T2DM: Type 2 Diabetes Mellitus
TG: Triglycerides
WC: Waist circumference
